# An exploration of research information security data affecting organizational compliance

**DOI:** 10.1016/j.dib.2018.11.002

**Published:** 2018-11-05

**Authors:** Sweden S. De Matas, Brendan P. Keegan

**Affiliations:** Veterans Health Administration Office of Research Oversight, United States

## Abstract

In this article, data collected from onsite assessments of federal healthcare research programs were reviewed and analyzed. 103 research programs were evaluated for adherence to federal and organizational information security requirements and the data clustered into three primary compliance groupings, technological, procedural, and behavioral. Frequency and cross-tabulation statistics were conducted and chi-square statistics used to test for associations.

**Specifications table**

**Table:** 

Subject area	Compliance, information security, behavior, research, technological, policy, education, employee decision-making, risk, organizations
More specific subject area	Research Information security compliance
Type of data	Tables, figures
How data was acquired	Onsite reports of research information security compliance reviews, Statistical Package for the Social Sciences (SPSS) Version 22 (Armonk, NY: IBM Corporation)
Data format	Filtered, analyzed
Experimental factors	Data obtained from research programs
Experimental features	Frequency, cross-tabulation and chi-square statistics
Data source location	Data represented federal healthcare research programs across the United States
Data accessibility	All the data are in this article

**Value of the data**

• Public availability and further analysis of this data will expand the literature regarding information security compliance including those specific factors that directly impact organizational risk mitigation strategy and employee adherence (e.g., employee decision-making).
• This analysis may further inform decisions surrounding routine technological and procedural resources for detecting and mitigating information security risk.
• The trends in this data will help inform information security compliance decisions regarding program development and employee behavior.
• This data provides the first comprehensive review of information security compliance in a research setting on an enterprise scale.

## 1 Data

**Fig. 1:**
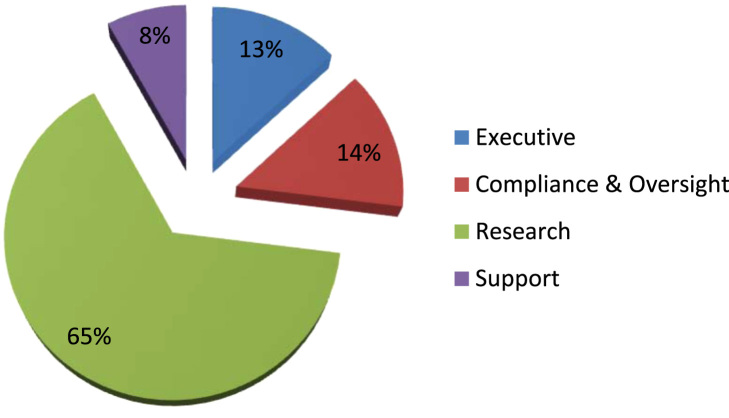
Participant descriptors.

The sample included data collected from onsite research information security compliance reviews completed by the Veterans Health Administration (VHA) Office of Research Oversight (ORO) from the year 2009 through 2017. The purpose of these reviews was to evaluate VHA research programs adherence to federal and organizational information security requirements. 103 research programs were evaluated with 10% of the sample size acquired from research programs located at VHA hospitals of lower complexity, 12% from research programs located at VHA hospitals of medium complexity, and 78% from research programs located at VHA hospitals of high complexity (see Table 1). Of the programs evaluated, over two thousand employees participated in the onsite reviews ranging from support to executive staff with the highest participation from the research program (see Fig. 1). Compliance and oversight staff accounted for 14% of employee participation and included Privacy Officers, Information Security Systems Officers (ISSOs), and Research Compliance Officers.

**Table 1:** Facility descriptors.

Research Programs Evaluated	VHA Hospital Complexity Level		Description
41	1a	HIGH	Highest patient volume and risk
			Teaching and research
			Contains level 5 Intensive Care Units (ICUs)
17	1b		Very high patient volume and risk
			Teaching and research
			Contains levels 4 and 5 ICUs
22	1c		High patient volume and risk
			Teaching and research
			Contains level 4 ICUs
11	2	MEDIUM	Medium patient volume and risk
			Some teaching and research
			Contains levels 3 and 4 ICUs
12	3	LOW	Low levels of patent volume and risk
			Little to no teaching and research
			Contains level 1 and 2 ICUs

Information collected during the onsite research information security compliance reviews were derived from in-depth interviews, document reviews, and physical evaluations of the research space including offices, laboratories, assigned clinical spaces, and server rooms. In addition, physical evaluations of certain data capable information technology (IT) equipment were completed as part of each review.

Noncompliance for each site was documented in a site-specific report, and the data contained in those reports compiled and subjected to statistical analysis. In addition, anecdotal evidences contained in reviewer notes relating to the reasons for the noncompliance were also qualitatively aggregated.

## 2 Experimental design, materials and methods

Onsite reports were reviewed and each finding of noncompliance placed in one of fifteen broad categories (see Table 2). Those categories were further distilled and the findings of noncompliance clustered based on similarity, and placed into seven primary groupings (Use of external information systems, management of research information, use of mobile and portable devices, ISSO reviews, privacy-related requirements, training, and reporting). The findings in each of the seven categories were then separated into three subcategories representing technological, procedural, and behavioral implications. For example, if an automated backup of research related data failed; the consequential finding was placed into the technological subcategory. Likewise, if the noncompliance was because of an erroneous policy or required form, that finding was placed in the procedural subcategory. Last, noncompliance as a direct consequence of an employee behavior such as the failure of research staff to properly store and/or transmit sensitive research data in compliance with established policy, the failure to report a research information security incident, or complete required training was relegated to the behavioral subcategory. The ensuing data are illustrated in Table 3, Table 4, Table 5, Table 6, Table 7.^1^

**Table 2:** Data categories, description of noncompliance and groupings.

Category	Findings of Noncompliance (for example)	Grouping	Subcategories
Documentation of System Interconnections	Erroneous or missing agreements	Use of External Information Systems	Technological Procedural Behavioral
Documentation of air-gapped networks	Erroneous or missing agreements		
External storage of sensitive information	Unauthorized offsite storage		
Inventory of externally owned equipment	Equipment use to process and store human subjects’ data not appropriately accounted		
Internal Storage of sensitive information	Pervasive permissions to protected health information	Management of Research Information	Technological Procedural Behavioral
Encryption of sensitive information during transmission	Lack of compliant encryption standards when transmitting human subjects’ data		
Authorization to transport sensitive information	Removal and transport of human subjects’ data without approval		
Authorized use of mobile systems	Use of mobile devices (e.g., laptop) to process and store human subjects’ data without approval	Use of Mobile and Portable Devices	Technological Procedural Behavioral
Encryption of mobile systems	Lack of compliant encryption standards when using human subjects’ data on mobile devices		
Encryption of removable media containing sensitive information	Lack of compliant encryption standards when using human subjects’ data on removable media (e.g., thumb drive)		
Authorized use of personal equipment	Use of personally owned equipment to process and store human subjects’ data without approval		
ISSO Review	Erroneous or missing required reviews	ISSO Review	Technological Procedural Behavioral
Privacy Related Requirements	Incorrect implementation of procedural requirements or noncompliance related (?)	Privacy Related Requirements	Technological Procedural Behavioral
Training	Missing required trainings	Training	Technological Procedural Behavioral
Proper reporting of research-related information security incidents	Deficient procedures for and/or reporting of human subjects’ research incidents	Proper reporting of research information security incidents	Technological Procedural Behavioral

**Table 3:** Noncompliance identified at research programs located at VHA hospitals of high (level 1a) complexity.

CPXITY	**EIS**			**MRI**			**MPD**			**IR**			**PR**			**TRNG**			**REP**		
	T	P	B	T	P	B	T	P	B	T	P	B	T	P	B	T	P	B	T	P	B
1a	0	1	1	0	0	0	0	0	1	0	1	1	0	1	1	0	0	0	0	0	0
1a	0	1	1	0	0	1	0	0	1	0	0	0	0	1	1	0	0	0	0	0	0
1a	0	1	1	0	0	1	0	0	0	0	0	0	0	1	1	0	0	1	0	0	0
1a	0	1	1	0	1	1	0	0	0	0	0	0	0	1	1	0	1	1	0	0	0
1a	0	1	1	0	0	1	0	0	1	0	1	1	0	1	1	0	0	1	0	0	0
1a	0	0	0	0	0	1	0	0	0	0	1	1	0	1	1	0	0	1	0	0	0
1a	0	0	0	0	0	1	0	0	0	0	1	1	0	1	1	0	0	0	0	1	0
1a	0	1	1	0	1	1	0	0	0	0	1	1	0	1	1	0	0	1	0	1	0
1a	0	1	1	0	0	1	0	0	1	0	0	1	0	1	1	0	0	0	0	0	1
1a	1	1	1	0	0	1	0	0	1	0	0	1	0	1	1	0	0	1	0	0	0
1a	0	0	1	0	0	1	0	0	1	0	1	1	0	1	1	0	0	0	0	1	0
1a	0	1	1	0	0	1	0	0	1	0	0	1	0	1	1	0	0	0	0	1	0
1a	0	1	1	0	0	1	0	0	1	0	1	1	0	1	1	0	0	0	0	1	0
1a	0	0	1	0	0	1	0	0	0	0	0	1	0	1	1	0	0	0	0	0	0
1a	0	1	1	0	0	1	0	0	1	0	1	1	0	1	1	0	0	0	0	1	0
1a	0	0	1	0	0	1	0	0	1	0	0	1	0	1	1	0	0	1	0	0	0
1a	0	1	1	0	0	1	0	0	1	0	1	1	0	1	1	0	0	0	0	0	0
1a	0	1	1	0	0	1	0	0	1	0	1	0	0	1	1	0	0	1	0	1	0
1a	0	0	1	1	1	1	0	0	1	0	0	1	0	1	1	0	0	0	0	1	0
1a	1	1	1	0	0	1	0	0	0	0	1	1	0	1	1	0	0	0	0	0	0
1a	0	1	1	0	0	1	0	0	0	0	1	1	0	1	1	0	0	0	0	0	1
1a	0	1	1	0	1	1	0	0	0	0	1	1	0	1	1	0	0	0	0	1	0
1a	0	1	1	0	0	1	0	0	0	0	1	1	0	1	1	0	0	0	0	0	0
1a	0	1	1	0	0	1	0	0	1	0	1	0	0	1	1	0	0	0	0	1	0
1a	0	0	1	0	0	1	0	0	1	0	1	1	0	1	1	0	1	0	0	1	0
1a	0	1	1	0	0	1	0	0	1	0	1	1	0	1	1	0	0	0	0	0	0
1a	0	1	1	0	0	1	0	0	1	0	1	1	0	1	1	0	0	1	0	1	0
1a	0	1	1	0	0	1	0	0	1	0	1	1	0	1	1	0	0	0	0	1	0
1a	0	1	1	0	0	1	0	0	1	0	0	1	0	1	1	0	0	0	0	0	0
1a	0	1	1	0	0	1	0	0	1	0	0	0	0	1	1	0	0	0	0	0	0
1a	0	1	1	0	0	1	0	0	1	0	0	1	0	0	0	0	0	0	0	1	0
1a	0	1	1	0	0	1	0	0	0	0	0	0	0	0	0	0	0	0	0	0	1
1a	0	1	1	0	0	1	0	0	1	0	0	1	0	1	1	0	0	0	0	0	0
1a	0	1	1	0	0	1	0	0	1	0	0	1	0	0	0	0	0	0	0	0	0
1a	0	0	0	0	0	1	0	0	1	0	0	1	0	0	0	0	0	0	0	0	0
1a	0	1	1	0	0	0	0	0	1	0	0	0	0	0	0	0	0	0	0	1	0
1a	0	1	1	0	0	1	0	0	1	0	0	0	0	0	0	0	0	0	0	0	0
1a	0	1	1	0	0	1	0	0	1	0	0	0	0	0	0	0	0	0	0	0	0
1a	0	1	1	0	0	1	0	0	1	0	0	0	0	1	1	0	0	0	0	0	0
1a	0	1	1	0	0	1	0	0	1	0	0	0	0	0	0	0	0	0	0	0	0
1a	0	1	1	0	0	1	0	0	1	0	0	0	0	1	1	0	0	0	0	1	0

Key (Applicable to Table 3, Table 4, Table 5, Table 6, Table 7): 0 – No finding; 1 – Finding of Non-compliance; EIS – Use of External Information Systems; MRI – Management of Research Information; MPD – Use of Mobile and Portable Devices; IR – ISSO Review; PR – Privacy Related Requirements; TRNG – Training; REP – Proper Reporting of Research Information Security Incidents; CPXITY – Facility Complexity; T – Technological; P – Procedural; B - Behavioral.

**Table 4:** Noncompliance identified at research programs located at VHA hospitals of high (level 1b) complexity.

CPXITY	**EIS**			**MRI**			**MPD**			**IR**			**PR**			**TRNG**			**REP**		
	T	P	B	T	P	B	T	P	B	T	P	B	T	P	B	T	P	B	T	P	B
1b	0	1	1	0	0	1	0	0	1	0	0	1	0	1	1	0	0	0	0	0	0
1b	0	0	1	0	0	1	0	0	1	0	0	1	0	0	0	0	0	0	0	0	0
1b	0	0	1	0	1	1	0	0	1	0	1	1	0	1	1	0	0	0	0	1	0
1b	0	1	1	0	0	1	0	0	1	0	0	1	0	1	1	0	0	0	0	1	0
1b	0	0	1	0	0	1	0	0	1	0	1	1	0	1	1	0	0	0	0	1	0
1b	0	0	1	0	0	1	0	0	1	0	1	1	0	1	1	0	0	0	0	0	0
1b	0	1	1	0	0	1	0	0	1	0	1	1	0	1	1	0	0	1	0	0	0
1b	0	0	1	0	0	1	0	0	0	0	1	1	0	1	1	0	0	1	0	1	0
1b	0	0	1	0	0	1	0	0	1	0	1	1	0	1	1	0	0	1	0	0	0
1b	0	1	1	0	0	1	0	0	1	0	1	1	0	1	1	0	0	0	0	0	0
1b	0	1	1	0	0	1	0	0	0	0	1	1	0	1	1	0	0	0	0	0	0
1b	0	0	1	0	0	1	0	0	1	0	0	0	0	1	1	0	0	0	0	1	0
1b	0	0	1	0	1	1	0	0	1	0	0	1	0	1	1	0	0	0	0	1	0
1b	0	1	1	0	0	1	0	0	1	0	0	1	0	0	0	0	0	0	0	0	0
1b	0	1	1	0	0	1	0	0	1	0	0	0	0	0	0	0	0	0	0	1	0
1b	0	1	1	0	0	1	0	0	0	0	0	0	0	0	0	0	0	0	0	1	0
1b	0	0	0	0	0	0	0	0	0	0	0	0	0	0	0	0	0	0	0	1	0

**Table 5:** Noncompliance identified at research programs located at VHA hospitals of high (level 1c) complexity.

CPXITY	**EIS**			**MRI**			**MPD**			**IR**			**PR**			**TRNG**			**REP**		
	T	P	B	T	P	B	T	P	B	T	P	B	T	P	B	T	P	B	T	P	B
1c	0	0	1	0	0	1	0	0	1	0	0	1	0	1	1	0	0	0	0	0	0
1c	0	0	0	0	0	1	0	0	1	0	0	1	0	1	1	0	0	0	0	0	0
1c	0	0	0	0	0	1	0	0	0	0	1	1	0	1	1	0	0	1	0	1	0
1c	1	1	1	0	0	1	0	0	1	0	1	1	0	1	1	0	0	0	0	1	0
1c	0	1	1	0	0	1	0	0	0	0	1	1	0	1	1	0	0	0	0	1	0
1c	0	1	1	0	0	1	0	0	1	0	1	1	0	1	1	0	0	0	0	1	0
1c	0	0	0	0	0	0	0	0	0	0	0	0	0	1	1	0	0	0	0	1	0
1c	0	0	1	0	0	0	0	0	1	0	0	1	0	1	1	0	0	1	0	1	0
1c	1	0	1	0	0	1	0	0	0	0	0	0	0	1	1	0	0	0	0	0	0
1c	0	0	0	0	0	1	0	0	0	0	1	1	0	1	1	0	0	0	0	0	0
1c	0	0	0	0	0	1	0	0	0	0	0	0	0	1	1	0	0	0	0	0	0
1c	0	0	0	0	0	1	0	0	0	0	0	1	0	1	1	0	0	1	0	1	0
1c	0	0	0	0	0	1	0	0	1	0	0	1	0	1	1	0	0	0	0	1	0
1c	0	0	0	0	0	1	0	0	1	0	1	1	0	1	1	0	0	0	0	1	0
1c	0	0	1	0	0	1	0	0	1	0	0	0	0	0	0	0	0	0	0	1	0
1c	0	1	1	0	0	1	0	0	1	0	0	0	0	0	0	0	0	0	0	0	0
1c	0	1	1	0	0	1	0	0	1	0	0	1	0	0	0	0	0	0	0	1	0
1c	0	1	1	0	0	1	0	0	1	0	0	0	0	0	0	0	0	0	0	0	0
1c	0	1	1	0	0	1	0	0	0	0	0	0	0	0	0	0	0	0	0	1	0
1c	0	1	1	0	0	1	0	0	1	0	0	1	0	0	0	0	0	0	0	1	0
1c	0	0	0	0	0	0	0	0	0	0	0	1	0	0	0	0	0	0	0	1	0
1c	0	1	1	0	0	1	0	0	0	0	0	0	0	0	0	0	0	0	0	0	0

**Table 6:** Noncompliance identified at research programs located at VHA hospitals of medium (level 2) complexity.

CPXITY	**EIS**			**MRI**			**MPD**			**IR**			**PR**			**TRNG**			**REP**		
	T	P	B	T	P	B	T	P	B	T	P	B	T	P	B	T	P	B	T	P	B
2	0	0	0	0	0	1	0	0	0	0	1	1	0	1	1	0	0	0	0	0	0
2	0	1	1	0	0	1	0	0	0	0	1	0	0	1	1	0	0	0	0	0	0
2	0	0	1	0	1	1	0	0	0	0	0	1	0	1	1	0	0	1	0	0	0
2	0	0	1	0	0	0	0	0	0	0	0	0	0	1	1	0	0	0	0	0	0
2	0	0	0	0	0	1	0	0	0	0	0	1	0	1	1	0	0	1	0	1	0
2	0	0	0	0	0	1	0	0	1	0	1	1	0	1	1	0	1	0	0	0	1
2	0	1	1	0	0	1	0	0	1	0	0	1	0	1	1	0	0	0	0	1	0
2	0	0	0	0	0	0	0	0	0	0	0	0	0	1	1	0	0	0	0	1	0
2	0	0	0	0	0	0	0	0	0	0	0	1	0	0	0	0	0	0	0	0	0
2	0	0	0	0	0	1	0	0	0	0	0	0	0	0	0	0	0	0	0	1	0
2	0	0	0	0	0	1	0	0	0	0	0	1	0	0	0	0	0	0	0	1	0

**Table 7:** Noncompliance identified at research programs located at VHA hospitals of low (level 3) complexity.

CPXITY	**EIS**			**MRI**			**MPD**			**IR**			**PR**			**TRNG**			**REP**		
	T	P	B	T	P	B	T	P	B	T	P	B	T	P	B	T	P	B	T	P	B
3	0	0	0	0	0	1	0	0	0	0	1	1	0	1	1	0	0	0	0	0	0
3	0	0	0	1	0	1	0	0	0	0	0	0	0	1	1	0	0	0	0	1	0
3	0	0	0	0	0	0	0	0	0	0	1	0	0	1	1	0	0	0	0	0	0
3	0	1	1	0	0	1	0	0	0	0	0	1	0	1	1	0	0	0	0	1	0
3	0	1	1	1	0	1	0	0	1	0	0	0	0	0	0	0	0	0	0	0	0
3	0	0	0	0	0	1	0	0	0	0	0	1	0	1	1	0	0	0	0	0	0
3	0	0	0	0	0	0	0	0	0	0	0	1	0	0	0	0	0	0	0	0	0
3	0	1	1	0	0	1	0	0	0	0	0	0	0	0	0	0	0	0	0	1	0
3	0	0	0	0	0	0	0	0	0	0	0	0	0	0	0	0	0	0	0	0	0
3	0	0	0	0	0	1	0	0	0	0	0	0	0	0	0	0	0	0	0	1	0
3	0	0	0	0	0	0	0	0	0	0	0	0	0	0	1	0	0	0	0	0	0
3	0	0	0	0	0	1	0	0	1	0	0	0	0	0	0	0	0	0	0	1	0

For statistical analysis, frequency and cross-tabulation statistics were conducted to describe the sample and check for coding errors. Chi-square statistics were used to test for associations between complexity and noncompliance for each area of interest. Significant associations were reported using unadjusted odds ratios (OR) with 95% confidence intervals (95% CI). Statistical significance was assumed at an alpha value of 0.05 and all analyses were conducted using the Statistical Package for the Social Sciences (SPSS) Version 22 (Armonk, NY: IBM Corporation).

Chi-square statistics found several significant differences in rates of noncompliance between the complexity groups. Research programs located at complex VHA hospitals were five times more likely (95% CI 1.25–19.93) to have procedural noncompliance with the use of external information systems versus research programs located at those VHA hospitals of lower complexity. Similarly, the trend was that research programs located at higher complex VHA hospitals were more likely to have higher rates of behavioral noncompliance versus those research programs located at VHA hospitals with a lower complexity in the categories of behavioral noncompliance associated with the use of external information systems (OR 15.46 [95% CI 3.68–64.95]), the management of research information (OR 6.17 [95% CI 1.43–26.56]), the use of mobile and portable devices (OR 11.00 [95% CI 2.24–53.95]), and the ISSO review of research projects (OR 4.40 [95% CI 1.21–15.98]). Higher levels of procedural noncompliance related to privacy related requirements were also observed in research programs located at more complex VHA hospitals versus those of lower complexity (OR 3.93 [95% CI 1.13–13.74]).

The single exception to the trend involved technological noncompliance related to the management of research information where research programs located at more complex VHA hospitals were less likely to have noncompliance versus those programs located at VHA hospitals with a lower complexity (OR 0.63 [95% CI 0.01–0.76]). No significant differences were observed between those research programs located at VHA hospitals of a medium complexity and those with a lower complexity in terms of noncompliance for any area. Frequencies and percentages associated with noncompliance for each area of interest and by complexity are in Table 8.

**Table 8:** Percentage of noncompliance.

Grouping	Area	Low Complexity	Medium Complexity	High Complexity	*p*-Value
Use of External Information Systems	Technology	0 (0.0%)	0 (0.0%)	4 (5.0%)	0.55
	Procedure	3 (25.0%)	2 (18.2%)	50 (62.5%)	0.006*
	Behavior	3 (25.0%)	4 (36.4%)	67 (83.8%)	< 0.001*
Management of Research Information	Technology	2 (16.7%)	0 (0.0%)	1 (1.3%)	0.09
	Procedure	0 (0.0%)	1 (9.1%)	6 (7.5%)	0.60
	Behavior	8 (66.7%)	8 (72.7%)	74 (92.5%)	0.02
Use of Mobile and Portable Devices	Technology	0 (0.0%)	0 (0.0%)	0 (0.0%)	–
	Procedure	0 (0.0%)	0 (0.0%)	0 (0.0%)	–
	Behavior	2 (16.7%)	2 (18.2%)	55 (68.8%)	< 0.001*
ISSO Review	Technology	0 (0.0%)	0 (0.0%)	0 (0.0%)	–
	Procedure	2 (16.7%)	3 (27.3%)	33 (41.3%)	0.20
	Behavior	4 (33.3%)	7 (63.6%)	55 (68.8%)	0.08
Privacy Related Requirements	Technology	0 (0.0%)	0 (0.0%)	0 (0.0%)	–
	Procedure	5 (41.7%)	8 (72.7%)	59 (73.8%)	0.10
	Behavior	5 (50.0%)	8 (72.7%)	59 (73.8%)	0.24
Training	Technology	0 (0.0%)	0 (0.0%)	0 (0.0%)	–
	Procedure	0 (0.0%)	1 (9.1%)	2 (2.5%)	0.39
	Behavior	0 (0.0%)	2 (18.2%)	15 (18.8%)	0.26
Proper Reporting of Research Information Security Incidents	Technology	0 (0.0%)	0 (0.0%)	0 (0.0%)	–
	Procedure	5 (41.7%)	5 (45.5%)	39 (48.8%)	0.89
	Behavior	0 (0.0%)	1 (9.1%)	3 (3.8%)	0.53

* *p*< 0.05.

By far, the highest rates of noncompliance occurred in the behavioral category, and observed across all areas of analysis (use of external information systems, management of research information, use of mobile and portable devices, ISSO reviews, privacy related noncompliance, training, and the proper reporting of research information security incidents). In addition, rates of procedural noncompliance associated with the proper reporting of research information security incidents were above 40% for research programs at all VHA hospital levels. Public availability and further review and analysis of this data will expand the literature regarding information security compliance including those specific factors that directly impact organizational risk mitigation strategy and employee adherence (e.g., employee decision-making) (Griffith, 2016; Durgin, 2007; Werlinger et al., 2008) [1], [2], [3]. The identified trends will help inform information security compliance decisions regarding program development 4 and employee behavior; (Guest, 2016; Whyte, 2016; Abed and Weistroffer, 2016; Haidt, 2013) [4], [5], [6], [7] and may further inform decisions surrounding routine technological and procedural resources for detecting and mitigating information security risk (Kayworth and Whitten, 2010; Bulgurcu et al., 2010; Haugh, 2017) [8], [9], [10].

## Footnotes

1 It should be noted that the data does not present a *total* number of instances of noncompliance in each *category* encountered. For example, if a facility was cited for noncompliance associated with the lack of proper encryption observed over multiple devices; a single finding represented that noncompliance in the onsite reports, rather than a finding written for each noncompliant device.
